# Unfocused shockwaves for osteoinduction in bone substitutes in rat cortical bone defects

**DOI:** 10.1371/journal.pone.0200020

**Published:** 2018-07-03

**Authors:** Marianne K. E. Koolen, Behdad Pouran, Fetullah C. Öner, Amir A. Zadpoor, Olav P. van der Jagt, Harrie Weinans

**Affiliations:** 1 Department of Orthopaedics, University Medical Centre Utrecht, Utrecht, the Netherlands; 2 Department of Biomechanical Engineering, Faculty of Mechanical, Maritime, and Materials Engineering, Delft University of Technology, Delft, The Netherlands; 3 Department of Orthopaedics, Elisabeth-TweeSteden Hospital, Tilburg, the Netherlands; 4 Department of Rheumatology and Clinical Immunology, University Medical Centre Utrecht, Utrecht, the Netherlands; Ohio State University, UNITED STATES

## Abstract

Bone substitutes are frequently used in clinical practice but often exhibit limited osteoinductivity. We hypothesized that unfocused shockwaves enhance the osteoinductivity of bone substitutes and improve osteointegration and angiogenesis. Three different bone substitutes, namely porous tricalcium phosphate, porous hydroxyapatite and porous titanium alloy, were implanted in a critical size (i.e. 6-mm) femoral defect in rats. The femora were treated twice with 1500 shockwaves at 2 and 4 weeks after surgery and compared with non-treated controls. The net volume of *de novo* bone in the defect was measured by microCT-scanning during 11-weeks follow-up. Bone ingrowth and angiogenesis in the bone substitutes was examined at 5 and 11 weeks using histology. It was shown that hydroxyapatite and titanium both had an increase of bone ingrowth with more bone in the shockwave group compared to the control group, whereas resorption was seen in tricalcium phosphate bone substitutes over time and this was insensitive to shockwave treatment. In conclusion, hydroxyapatite and titanium bone substitutes favour from shockwave treatment, whereas tricalcium phosphate does not. This study shows that osteoinduction and osteointegration of bone substitutes can be influenced with unfocused shockwave therapy, but among other factors depend on the type of bone substitute, likely reflecting its mechanical and biological properties.

## Introduction

Bone is an adaptive tissue, which is able to regenerate in a self-regulated process. Unfortunately, the capacity of bone regeneration is limited. In certain cases a bone defect may not heal and a non-union develops [1]. The treatment of such cases is a clinical challenge and sometimes demands the use of a synthetic bone substitute material, due to the limited availability and possible complications of autografts or allografts [2].

Popular bone substitutes in the clinic are porous tricalcium phosphate (TCP) and hydroxyapatite (HA), which are both biocompatible, biodegradable, and meant to be osteoconductive as they are made of materials that are part of the natural bone matrix [3]. Titanium and titanium alloys are well-established biocompatible materials in orthopaedic practice that could be employed as bone substitutes as well and provide immediate mechanical support [4].

The major drawback of bone substitutes is their limited osteoinductive properties [5]. An alternative method to enhance bone regeneration in bone substitutes might be the application of extracorporeal shockwaves (ESW). ESW are high-energy acoustical waves that showed stimulation of angiogenesis and osteogenesis in animal experiments [6, 7]. ESW are clinically used for the treatment of non-unions [8], and other musculoskeletal conditions [9]. Unfocused extracorporeal shockwaves (UESW) differ from original ESW in that they are produced in a non-focused or more parallel bundle, which enables treatment of larger (skeletal) areas [10] and thereby being optimally suited for large bone defects. In previous studies, distinct osteoinductive effects of bone were shown with UESW in healthy and osteoporotic rats [11, 12]. Shockwave therapy can be applied directly after surgical placement of a bone substitute and thereby enhance osteoinduction or osteointegration.

In the current study we examined if treatment with UESW of TCP, HA or titanium as a bone substitute material, stimulates bone ingrowth.

## Materials and methods

### Study design

**Fig 1:**
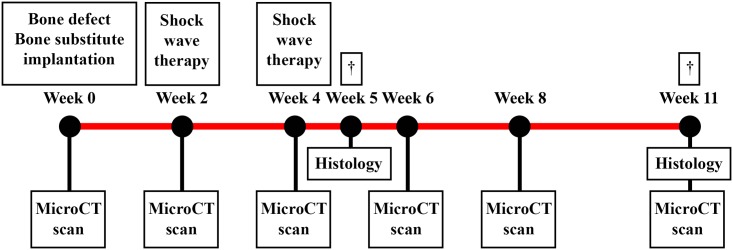
Schematic set-up of the study. An overview of all procedures and measurements performed during the eleven week follow-up.

Male Wistar rats (Charles River, Sulzfeld, Germany) were housed in pairs under strict supervision in the animal facility of the UMC-Utrecht. Animals received standard food pellets and water *ad libitum* and were kept under climate-controlled conditions (21°C; 12h light/ 12h darkness). At the age of 16 weeks and after 7 days of acclimatization (weight = 340–370 gram), a 6-mm critical-sized segmental bone defect was created in the right femur of each rat.[4] Subsequently, one of the three bone substitutes; TCP, HA, or titanium was implanted in the defect. Two and four weeks after the implantation, half of the bone substitutes were treated with 1.500 unfocused electrohydraulically generated shockwaves (Orthowave 180c; MTS medical, Konstanz, Germany). Rats were euthanized after 5 or 11 weeks with an overdose of barbiturates (phenobarbital; 200 mg/kg body weight, TEVA Pharma, Haarlem, The Netherlands) and the bones were analysed with micro-computed tomography (microCT) and histology (Fig 1). These end points were chosen as we want to find an explanation for any differences in bone formation directly after the shockwaves were applied (five weeks) and after a longer amount of time. After eleven weeks we would expect a significant difference in bone formation in this model if there would be any difference [13, 14]. The protocol was approved by the animal ethics committee of the institution (DEC Utrecht, http://dx.doi.org/10.17504/protocols.io.pv5dn86, S1 File) in accordance with the national laws on animal experiments.

Each bone substitute group consisted of 20 animals, with 16 animals in an 11-week follow-up protocol of which 8 were in an UESW-treated group and 8 served as non-treated controls, and 4 animals in a 5-week follow-up protocol, used for histology at this intermediate time point (2 UESW-treated and 2 control).

### Surgical procedure

The surgeries were performed aseptically under general anesthesia (1–3.5% isoflurane, AST-Farma, Oudewater, The Netherlands). Briefly, the right hind leg was exposed and a 23 mm long polyether ether ketone plate was fixed to the anterolateral plane of the femur.[4] A 6-mm cortical bone segment was removed with a wire saw using a saw guide (RISystem, Davos, Switzerland). Subsequently, a TCP, HA or titanium bone substitute was implanted press-fit into the defect. Fascia and skin were sutured in layers using Vicryl Rapide 5–0. Subcutaneous pain medication (buprenorphine, 0.05 mg/kg body weight, AST-Farma, Oudewater, The Netherlands) was given pre-operatively and twice a day for the following three days to both UESW and control animals. Before surgery, rats received a single dose of antibiotics (enrofloxacin; 5mg/kg body weight, Bayer, Mijdrecht, The Netherlands).

### Bone substitutes

**Fig 2:**
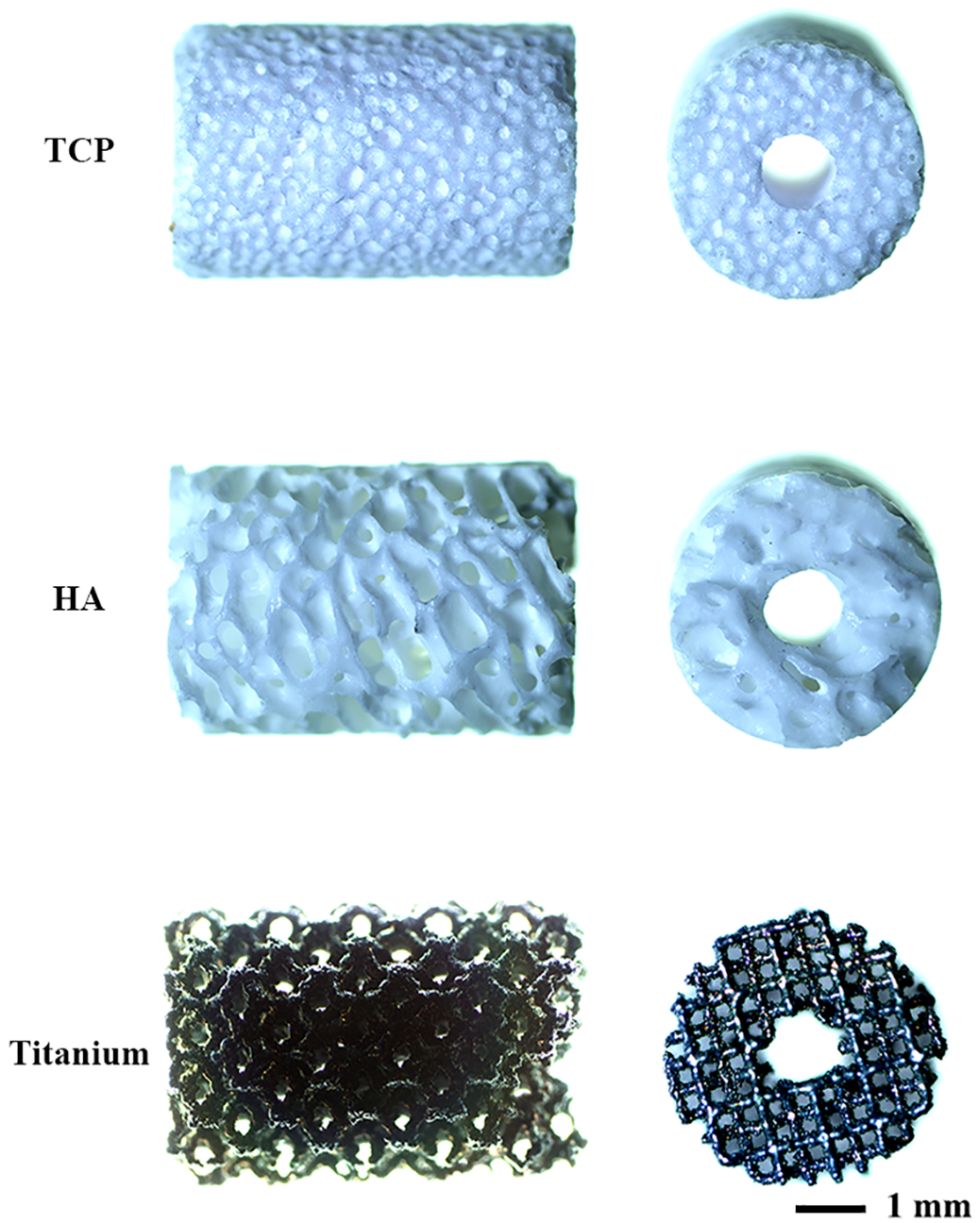
The three different bone substitutes. Tricalcium phosphate (TCP), hydroxyapatite (HA) and titanium bone substitutes were implanted in a 6-mm critical-sized segmental bone defect.

The three porous bone substitutes β-tricalcium phosphate (TCP), hydroxyapatite (HA) and titanium (titanium) were cylinders with a height of 6 mm, outer diameter of 4 mm, and inner diameter (endosteal canal) of 1 mm (Fig 2). TCP was produced with a 100–500 μm pore size and 70% porosity (ChronOS^™^, Synthes, Oberdorf, Switzerland) and HA with a 100–1500 μm pore size and 45–85% porosity (Endobon^®^, Biomet, Warsaw, Indiana). Titanium was produced from Ti6Al4V-ELI powder (ASTM B348, grade 23) using direct metal printing (DMP, ProX DMP 320, 3D Systems Layerwise, Leuven, Belgium). The porous architecture was based on a dodecahedron unit cell with a 240–730 μm pore size and 85% porosity. All titanium implants underwent a post-production alkali-acid-heat treatment [15].

### Unfocused shockwave therapy

Two weeks after the surgery, half of the bone substitutes were treated with 1500 unfocused electrohydraulically generated shockwaves, which are not radial shockwaves, but defocused shockwaves. These were applied at an energy level of 10 kV with an energy flux density of 0.3 mJ/mm2 (Orthowave 180c; MTS medical, Konstanz, Germany). Both number and energy setting of the shockwaves were chosen based on previous experiments [11, 12]. With the use of unfocused shockwaves, larger skeletal areas can be treated, which also enables application in large bone defects. Shockwave treatment was performed two weeks after placement of the bone substitutes as we believed that immediate shockwave treatment would interfere with the bone regeneration due to the operative procedure. After administration of pain medication, animals were placed under general anesthesia on their left dorsal-lateral side to treat the right leg. The hind leg was shaved and an ultrasonic gel was applied as coupling media between the applicator and the skin. The shockwave head was positioned at the anteromedial side of the hind leg and moved 180° around the mid-diaphysis of the femur. The focal area of the shockwave is 3.8 cm^2^ as to ensure the bone defect with the bone substitute as well as the distal and proximal femora are treated. Two weeks later the treatment was repeated, as we had seen in these previous experiments that the effect on bone formation subsided after two weeks [12, 16]. Another treatment could again trigger bone formation. On top of that, it is clinically known that non-unions treated with these kind of shockwaves make fusion after one or two treatments [8]. Control animals were not treated with UESW. Subcutaneous pain medication was given twice a day for three days.

### MicroCT scanning

For the three types of bone substitutes, pore size, strut thickness, and porosity were determined by microCT scanning. To measure bone ingrowth, repetitive *in vivo* microCT scanning of the femora were obtained under general anesthesia (1–3.5% isoflurane) from all animals at 0, 2, 4, 6, 8 and 11 weeks after the implantation. If UESW was applied, the microCT scanning was done in the same anesthesia session (at 2 and 4 weeks). In supine position, the hind leg of the rat was fixed, allowing scanning of the femur with microCT (scan time of 3 minutes, voxel size of 42 μm^3^, tube voltage of 90 kV, tube current of 180 μA, Quantum FX; PerkinElmer, Waltham, MA, USA).

**Fig 3:**
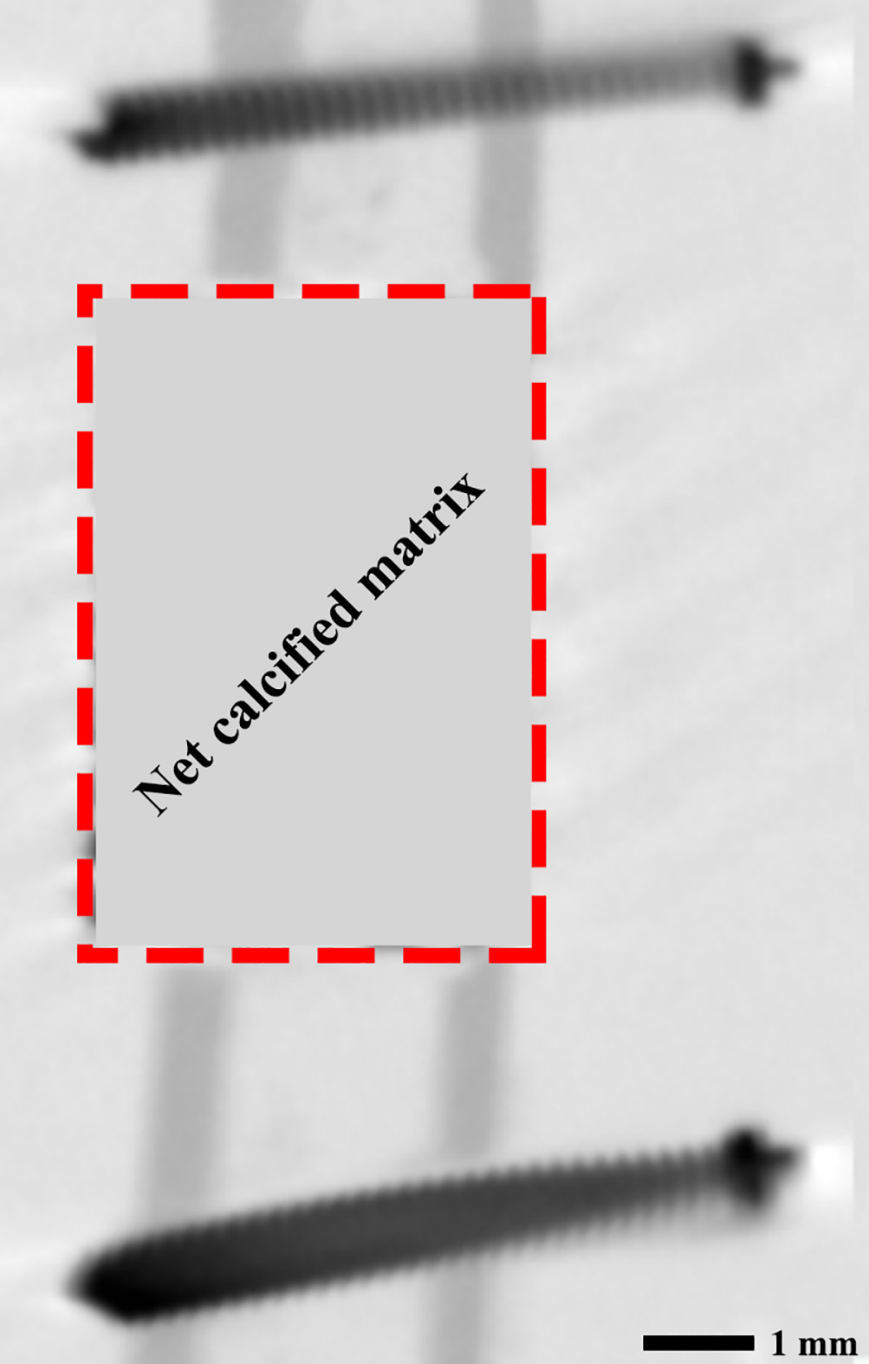
MicroCT analysis. The red dotted area is the region of interest of microCT measurements.

From all datasets, net volume change in the calcified matrix in the 6-mm defect site (region of interest, Fig 3) was determined using ImageJ (NIH, Redwood Shores, CA, USA). Since HA, TCP, and bone have approximately the same density, the scan results could not be directly translated into bone loss or bone formation. Therefore, the net volume of the calcified matrix (in mm^3^) was represented as the sum of newly formed bone plus the bone substitute (HA or TCP), which may have resorbed in time. As the region of interest only contains calcified matrix or soft tissue, the net volume fraction of the calcified matrix within that region could be determined from the mean grey value. The volume fractions of the calcified tissue in the region of interest were measured over time and compared to the initial volume fraction at the baseline.

### Histological evaluation

Histological evaluation was performed on two femora per group for both 5-weeks and 11-weeks follow-up groups. We used this small amount of animals only to describe the histology to find a possible explanation about the process of bone formation. Samples from the 11-week group were chosen to represent the mean of the whole group based on the net volume change of the calcified matrix determined earlier in the microCT. The samples were kept in a 4% neutral formalin buffered solution for 1 week. Titanium samples were dehydrated in ethanol series (70–100%) and embedded in methyl methacrylate. Sections in the coronal plane (thickness ~20 μm) were obtained using a diamond saw (Leica SP1600; Leica microsystems, Son, The Netherlands) and stained with 1% methylene blue (Sigma-Aldrich, Zwijndrecht, The Netherlands) and 0.3% basic fuchsin solution (Sigma-Aldrich, Zwijndrecht, The Netherlands) to stain bone pink and fibrous tissue blue. Serial sections (across the middle) were then screened for bone formation and bone-implant contact. HA and TCP samples were decalcified in 10% EDTA in phosphate buffered saline solution (pH 7.4) for 8 weeks and were dehydrated in graded ethanol solutions (70–100%) and xylene before embedding in paraffin. From the paraffin-embedded samples, sections of ~6 μm were obtained using a microtome (Microm HM340E; Thermo Fischer Scientific, Waltham, MA, USA). Overall appearance and new bone formation through serial sections (across the middle) were evaluated using H&E staining (Sigma-Aldrich, Zwijndrecht, The Netherlands).

An endothelial cell marker that indicates vessel-structures was determined by CD34 staining. Briefly, proteolysis mediated antigen retrieval was performed by 30 minutes incubation at 37° with 0.1% trypsin (Sigma-Aldrich, Zwijndrecht, The Netherlands). Endogenous peroxidase activity was blocked by incubating samples for 10 minutes in 0.3% H2O2. After blocking with 5% PBS-BSA for 30 minutes at room temperature, CD34 antibody was incubated with 10% normal goat serum for 1 hour at room temperature (1:200, AF4117; R&D Systems, Oxon, United Kingdom). After washing steps, all samples were incubated in 1% PBS-BSA with a HRP labelled secondary antibody (1:200, Dako, P0449) for 30 minutes at room temperature. The labelling was visualized with diaminobenzidine as substrate. Sections were then counterstained with haematoxylin.

Vascularization was also examined at 5-weeks follow-up with the use of a silicone injection compound with radiopaque contrast, next to the CD34 staining. For the 5-weeks follow-up group (2 animals per group), a catheter was placed under general anesthesia (1–3.5% isoflurane in oxygen) in the aorta abdominalis at the end point. First, 50 ml of 0.9% normal saline and then 100 ml of Papaverine solution (Sigma-Aldrich, Zwijndrecht, The Netherlands) was injected followed by 50 ml of 0.9% normal saline and 100 ml 10% of 4% neutral formalin buffered solution. After injection of a lead chromate radiopaque contrast agent (Microfil MV-120; Flow Tech, Carver, MA, USA), the compound was polymerized overnight at 4 °C. The next day, femora were removed and immersed in 4% phosphate-buffered paraformaldehyde.

### Statistical analysis

The data are presented as means and standard deviation unless otherwise indicated. In the analysis of the results of the microCT scanning, a mixed model was used to test for statistically significant differences between the UESW-treated and control bone substitutes (each bone substitute), while correcting for time effect using time as a random factor (SPSS 21.0 software IBM, Armonk, NY, USA). In addition, every time point for each bone substitute was analysed by *t*-tests. A *p*-value less than 0.05 was considered to indicate a statistically significant difference.

## Results

Animals had an average weight of 414 g (SD 56 g) at the time of implantation and during follow-up, there was an average increase of 30 g (SD 21 g) after 5 weeks and 56 g (SD 17) after 11 weeks and no significant differences in weight between UESW or control or between the bone substitute groups. One animal in the 11-weeks follow-up group (TCP) died due to anesthesia problems. No differences in physical activity were observed before and after treatment between the UESW-treated and control legs. No adverse events were seen in both treated and untreated legs.

### MicroCT analysis

TCP had an average strut size of 143.9 μm (SD 14.8), an average pore size of 267.9 μm (SD 85.0 μm), and a porosity of 43%. HA had an average strut size of 182.5 μm (SD 12.4), an average pore size of 365.2 μm (SD 48.1 μm), and a porosity of 67%. Porous titanium had an average strut size of 210.5 μm (SD 0.2), an average pore size of 243.9 μm (SD 0.4 μm), and a porosity of 79%. The changes in net calcified matrix after 11 weeks were overall significantly different between the three bone substitutes (*p* = 0.029) and over time (*p* < 0.001).

**Fig 4:**
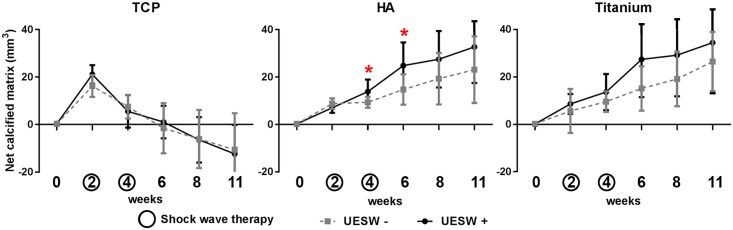
MicroCT results. Net volume change of the calcified matrix over time in TCP, HA and titanium bone substitutes. Lines present UESW treated (solid line) and untreated (dotted line) averages with standard deviation. * indicates p<0.05.

TCP bone substitutes showed an increase in net calcified matrix in the first two weeks after implantation (Fig 4, TCP), followed by continuous decrease in net calcified matrix within the ROI up to a net loss of 11.4 mm^3^ (SD 13.6) calcified matrix at end point 11 weeks after implantation. No difference in net calcified matrix was observed between the UESW-treated and non-treated controls during the 11-week follow-up period (mean difference 0.74, CI -4.91–6.39, *p* = 0.795).

HA bone substitutes showed continuous increase in net calcified matrix over time (Fig 4, HA) with a difference between the UESW-treated and control animals, although over time no statistically significant level was reached (mean difference 5.11 mm^3^, CI 0.4261–10.6525, *p* = 0.070). Per time point *t*-tests, however, did reach significance at 4 and 6 weeks. At 4 weeks, the net calcified matrix was 13.8 mm^3^ (SD 5.1) in UESW and 9.3 mm^3^ (SD 2.3) in non-treated controls (*p* = 0.039) and at 6 weeks, the net calcified matrix was 24.8 mm^3^ (SD 9.7) in UESW and only 14.7 mm^3^ (SD 6.4) in non-treated controls (*p* = 0.028). At end point, the net calcified matrix was 32.7 mm^3^ (SD 15.2) in the UESW-treated animals versus 23.1 mm^3^ (SD 5.0) in non-treated animals; an increase of 42% after shockwave therapy.

Titanium bone substitutes showed the highest increase in bone formation over time (Fig 4, Titanium) with 34.5 mm^3^ (SD 21.4) for treated animals compared to 26.5 mm^3^ (SD 12.5) for non-treated animals at 11 week after implantation. During follow-up, the average bone formation was higher in the UESW-treated animals as compared to non-treated controls at each time point, but this was not statistically different at any given time point nor in the mixed model over time (*p* = 0.138) with a mean difference of 6.23 mm^3^ (CI -2.04–14.50). The highest mean difference was noticed at 6 weeks with a mean difference of 12.3 mm^3^ (CI 5.8–18.9, *p* = 0.081).

### Ex vivo microCT analysis and histology

**Fig 5:**
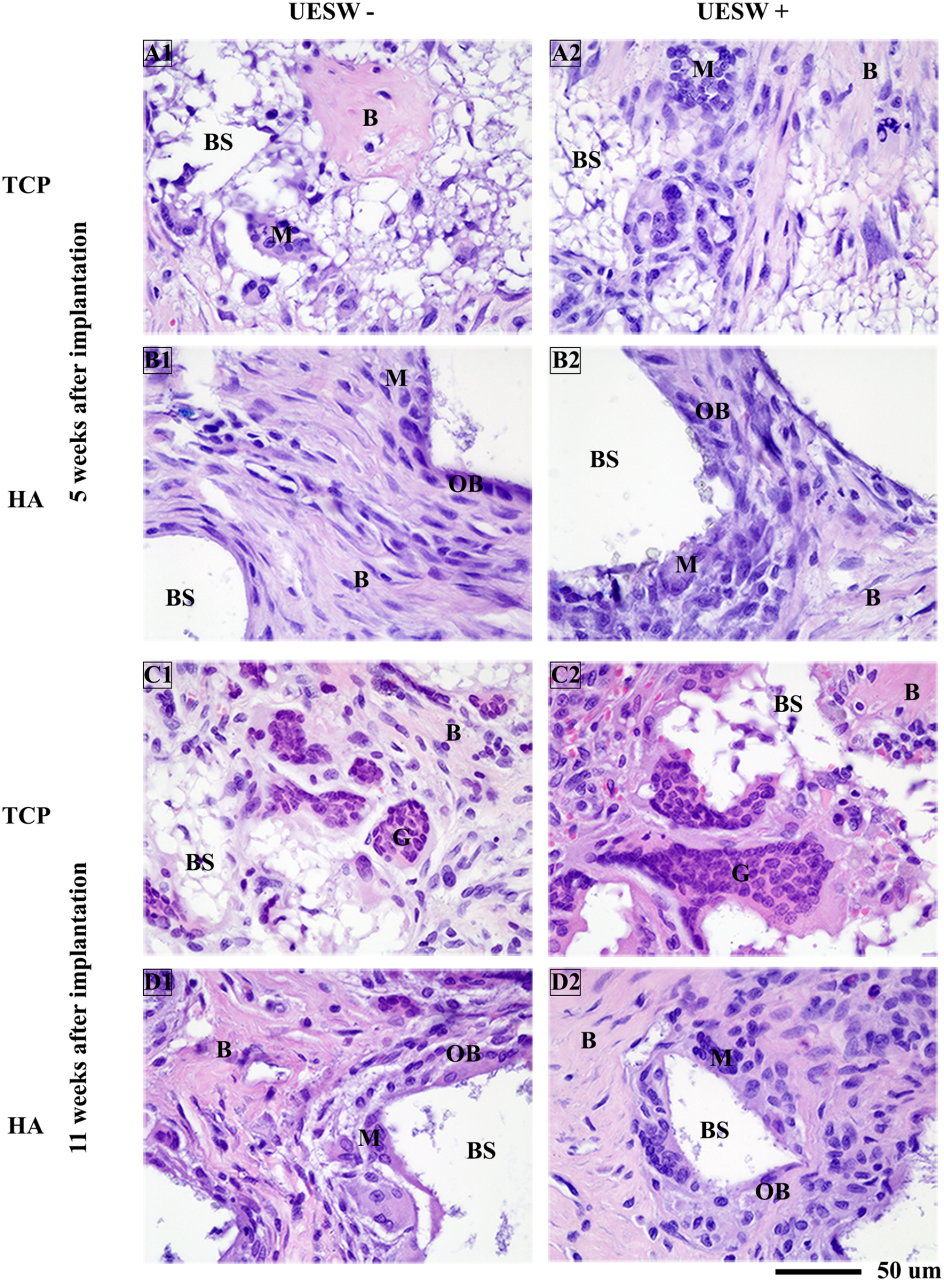
Histology of TCP and HA bone substitutes. Representative pictures of H&E staining taken from the middle of the scaffold. B = bone, BS = bone substitute, G = giant cells, M = macrophages, OC = osteoclasts, OS = osteoblasts, UESW = unfocused extracorporeal shockwaves.

**Fig 6:**
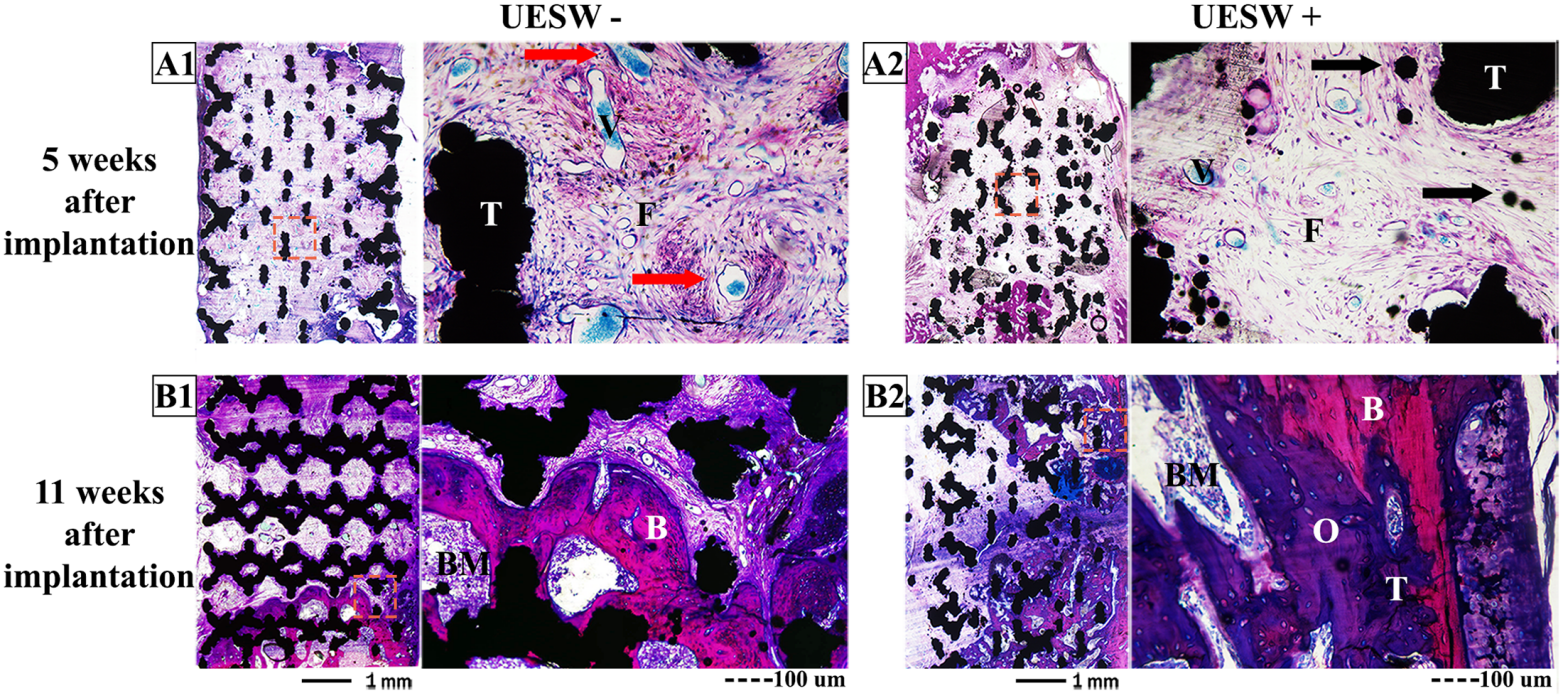
Histology of titanium bone substitutes. Overviews and zoomed (red box) pictures of titanium implants stained with basic fuchsin and methylene blue of sections 5 weeks (A) and 11 weeks (B) after implantation. Black arrows indicate loose titanium particles. Red arrows indicate blood vessels, which are filled with blue Microfil 5 weeks after implantation. B = bone, BM = bone marrow, F = fibrous tissue, O = osteoid, T = titanium, UESW = unfocused extracorporeal shockwaves.

Histology at five weeks and at the end point (week 11) confirmed an increase in bone formation for the UESW-treated HA and titanium bone substitutes. Resorption of TCP with foreign body giant cells was clearly visible in both treated and non-treated samples (Fig 5C). Both TCP and HA samples showed macrophages around the bone substitute material after five weeks and in HA also still some after eleven weeks, mostly after UESW as compared to the non-UESW controls (Fig 5A & 5B). HA samples also showed osteoblast after five weeks and after eleven weeks (Fig 5B & 5D). The HA bone substitutes appeared somewhat disrupted after UESW, while untreated substitutes still maintained their original architecture (data not shown). The UESW-treated titanium bone substitutes also seemed to have some loose titanium particles (Fig 6A2). Both treated and untreated samples showed large amounts of fibrous tissue inside the implants, which was even clearer in the untreated controls (Fig 6A). At end point of the study (11 weeks after implantation), bone ingrowth was clearly noticed in the titanium pores with clusters of osteoblasts, where the newly formed bone was in close contact with the titanium bone substitute (Fig 6B). The UESW-treated samples still showed considerable osteoid, indicating that maximum bone ingrowth was not reached yet after 11 weeks (Fig 6B2).

**Fig 7:**
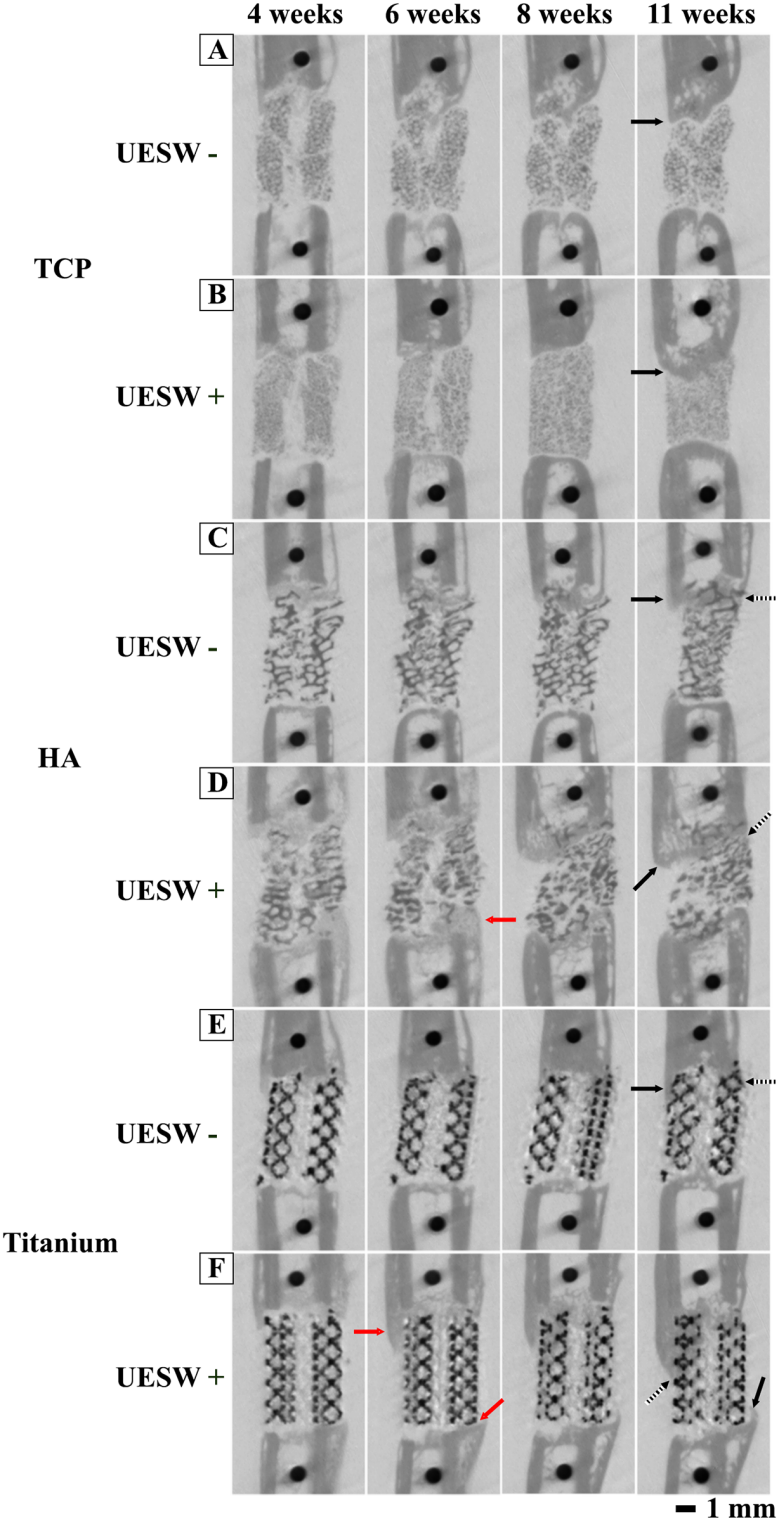
Representative longitudinal microCT scans of the femur illustrating the bone regeneration process. In HA and Titanium UESW treated bone substitutes there was already a raise of bone formation (red arrows) just after treatment compared to controls. Bone formation is observed outside (solid arrows) and inside (dotted arrows) bone substitutes. Red arrows indicate a raise of bone formation just after shockwave treatment. UESW = unfocused extracorporeal shockwaves. A = TCP UESW -, B = TCP UESW +, C = HA UESW -, D = HA UESW +, E = Titanium UESW -, F = Titanium UESW +.

MicroCT reconstructions over time showed most bone formation at the proximal side of the defect, which was confirmed with bone formation observed from histology (Fig 7). Most bone was formed at 6 weeks, 2 weeks after the second UESW treatment, in and around HA and titanium bone substitutes (Fig 7D & 7F). Bone formation in TCP was outside the bone substitute (Fig 7A & 7B), whereas in HA and titanium more bone was growing over time inside the bone substitute (Fig 7C–7F). None of the defects were completely bridged.

**Fig 8:**
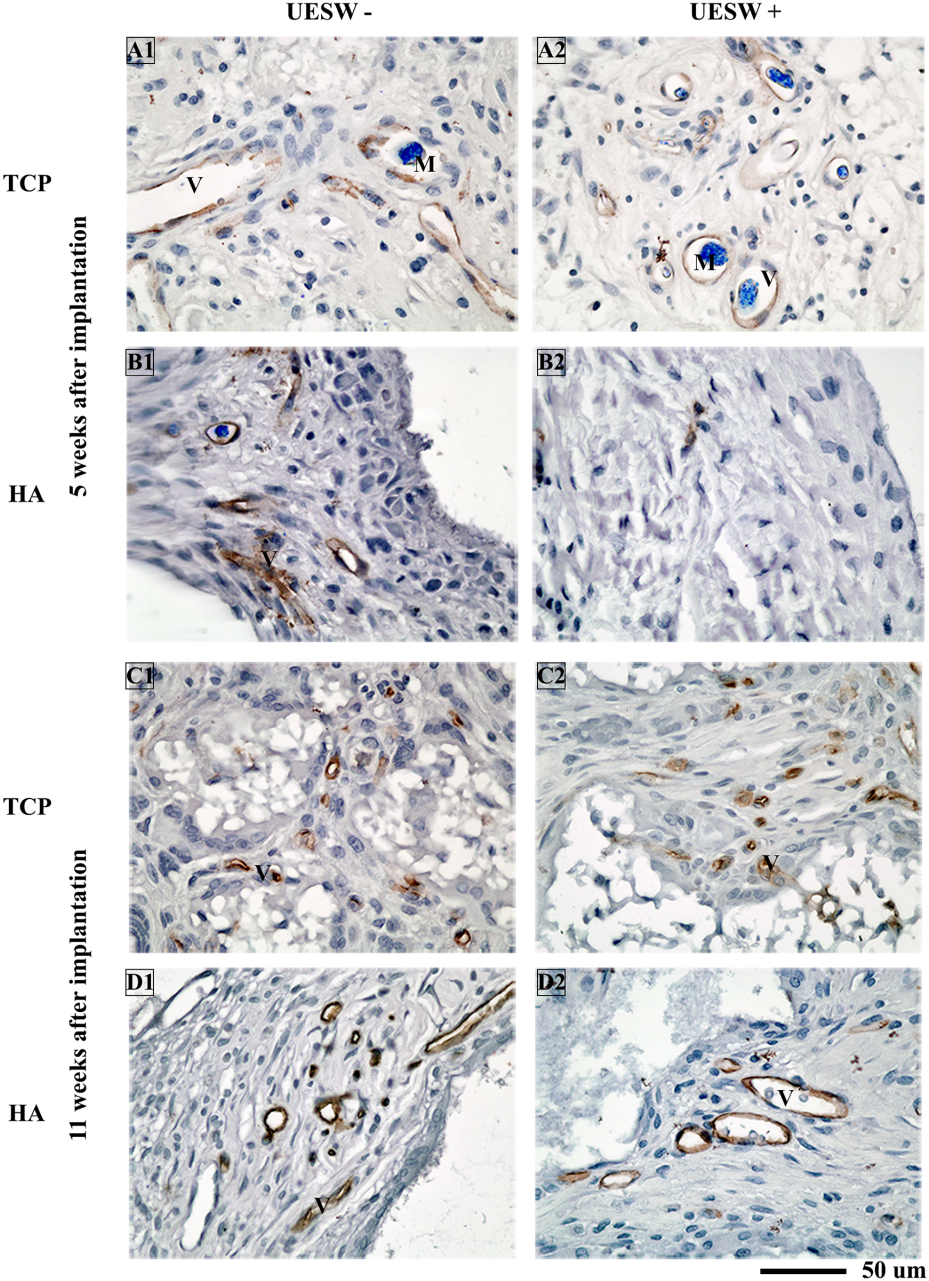
Histology of TCP and HA bone substitutes. Representative pictures of CD-34 staining (brown) taken from the middle of the scaffold. Blue indicates vessels by Microfil which was only used at end point 5 weeks after implantation. M = Microfil, UESW = unfocused extracorporeal shockwaves, V = vessel.

Vascularisation did not show any difference between treated and untreated TCP bone substitutes with respect to CD-34 staining (Fig 8A & 8C). The UESW treated HA samples showed less vessel formation after five weeks (Fig 8B1 & 8B2). On inspection, it seemed like less Microfil was found after shockwave therapy in the titanium substitutes compared to the non-shockwave treated titanium bone substitutes (Fig 6A1 & 6A2). As only two animals per group were used for histology and Microfil, no conclusions can be drawn using these methods.

## Discussion

The current study provided, for the first time, that UESW can improve bone regeneration in bone substitutes. Furthermore, this study shows that the effects of UESW depend on the type of the bone substitute. Both HA and titanium bone substitutes showed a consistent effect of shockwave therapy on bone formation, while the TCP bone substitutes did not respond to shockwave treatment.

It should be emphasized that the primary outcome in this study was the microCT analysis to determine the amount of bone change in the bone substitutes. There were only 8 animals per group with a rather large variation in bone formation at 11 weeks follow-up within each group. Although we consider the differences between UESW and control as (clinically) relevant, the sample size and variation provided *p*-values of 0.070 and 0.138, for HA and titanium respectively in the mixed models (variables time and group). Titanium bone substitute had most bone ingrowth from all tested bone substitutes. This was also observed in an earlier study [4], and might be related to high stiffness and mechanical stability of the titanium scaffold leading to an environment that stimulates bone growth [17]. It could also be due to its relatively high porosity in comparison to TCP or HA [18].

Interestingly, the positive effect as a consequence of UESW in the HA and titanium bone substitutes was the highest shortly after the second treatment. This direct effect of shockwave therapy was described before in other animal models [11]. However, the bone gaining effect did not continue until the end point of this study at 11 weeks after implantation of the bone substitutes. In an earlier study, bisphosphonates prevented subsequent resorption, causing a larger long-term anabolic effect [12]. Another intriguing observation was that the amount of blood vessels shortly after treatment appeared somewhat lower in shockwave treated HA and titanium samples. This might be due to the different types of tissue which were present at that time point inside the defect or this appearance might be incorrect due to the low number of animals that were used.

Bone formation around the TCP implants was not affected by UESW. Maybe TCP resorbs too fast over the time period of 11 weeks, which might be due to the foreign body reaction [19]. This resorption might overwhelm the anabolic effects of UESW. Another explanation could be that TCP has a lower porosity making it more difficult for the bone to grow in, thereby obscuring the UESW trigger [18].

It seems likely that differences in bone formation after shockwave therapy between the bone substitutes are due to a combination of biological and mechanical responses of the materials [20, 21], among other factors. Most of the energy of the UESW is taken up at the interfaces where bone and bone substitute join with different elastic moduli [9, 20]. Although the effects of UESW on HA and titanium bone substitutes were variable, it provided an average increase in bone ingrowth of 36% relative to the non-UESW treated animals, which is clinically relevant. Other important clinical factors for bone healing like the mechanical strength of the bone formed were unfortunately not tested but we do know that the more bone is formed, the higher the mechanical strength is [14]. Currently, bone substitutes are often used in osteoporotic fractures for example at the site of the tibia plateau, distal tibia, calcaneus and distal radius fractures [3]. Furthermore, stimulation of bone ingrowth and regeneration might be welcome in clinical situations with the use of trabecular titanium, e.g. in large acetabular or femoral condyle defects in complicated hip and knee revision arthroplasty. In this case it might be favourable to use unfocused, or also called defocused, shockwaves, as we did. Although it is not clear what the effect would be with the use of focused shockwaves. However, with the use of unfocused shockwaves larger skeletal areas can be treated, which enables application in these bone defects treated with bone substitutes. Further research needs to be performed to investigate these differences. The same accounts for the frequency and intensity; other settings might influence the results.

Shockwave therapy appeared to be safe without side effects apart from transient local redness and petechiae. However, shockwave therapy can be painful, and local or systemic anesthesia is required. For many applications UESW could be applied during primary surgery when a bone substitute is being placed. Moreover, a way to standardise the application of shockwaves should be established, which would probably reduce the great variation in outcome noticed in the current study.

To conclude, we showed that UESW applied in the post-operative phase of HA or titanium bone substitutes stimulate bone formation and thereby might improve fixation and stability of a bone substitute. As such, UESW might provide faster rehabilitation with earlier load bearing of the operated site. Furthermore, the clinical application of UESW is relatively safe. The current results justify further research to test UESW in a larger and more relevant model or in clinical settings.

## Supporting information

S1 File ARRIVE Guidelines Checklist. (PDF)
